# Ecology of bacteria in the human gastrointestinal tract—identification of keystone and foundation taxa

**DOI:** 10.1186/s40168-015-0107-4

**Published:** 2015-10-12

**Authors:** Pål Trosvik, Eric Jacques de Muinck

**Affiliations:** Centre for Ecological and Evolutionary Synthesis, Department of Biosciences, University of Oslo, P.O. Box 1066, Blindern, NO-0316 Oslo Norway

**Keywords:** Community ecology, Biotic interactions, Time series analysis, Keystone species, Foundation species, Limiting similarity

## Abstract

**Background:**

Determining ecological roles of community members and the impact of specific taxa on overall biodiversity in the gastrointestinal (GI) microbiota is of fundamental importance. A step towards a systems-level understanding of the GI microbiota is characterization of biotic interactions. Community time series analysis, an approach based on statistical analysis of changing population abundances within a single system over time, is needed in order to say with confidence that one population is affecting the dynamics of another.

**Results:**

Here, we characterize biotic interaction structures and define ecological roles of major bacterial groups in four healthy individuals by analysing high-resolution, long-term (>180 days) GI bacterial community time series. *Actinobacteria* fit the description of a keystone taxon since they are relatively rare, but have a high degree of ecological connectedness, and are positively correlated with diversity both within and between individuals. *Bacteriodetes* were found to be a foundation taxon in that they are numerically dominant and interact extensively, in particular through positive interactions, with other taxa. Although community structure, diversity and biotic interaction patterns were specific to each individual, we observed a strong tendency towards more intense competition within than between phyla. This is in agreement with Darwin’s limiting similarity hypothesis as well as a published biotic interaction model of the GI microbiota based on reverse ecology. Finally, we link temporal enterotype switching to a reciprocal positive interaction between two key genera.

**Conclusions:**

In this study, we identified ecological roles of key taxa in the human GI microbiota and compared our time series analysis results with those obtained through a reverse ecology approach, providing further evidence in favour of the limiting similarity hypothesis first put forth by Darwin. Larger longitudinal studies are warranted in order to evaluate the generality of basic ecological concepts as applied to the GI microbiota, but our results provide a starting point for achieving a more profound understanding of the GI microbiota as an ecological system.

**Electronic supplementary material:**

The online version of this article (doi:10.1186/s40168-015-0107-4) contains supplementary material, which is available to authorized users.

## Background

The complex microbial ecosystem of the gastrointestinal (GI) tract is important in human health and development [1]. One important step towards a systems-level understanding of the GI microbiota is characterization of biotic interactions. Different microbial taxa do not influence ecosystem processes equally, and determining the ecological roles of community members and the impact of specific taxa on overall biodiversity is of fundamental importance [2]. As we have previously presented [3], community time series analysis, an approach based on statistical analysis of changing population abundances within a single system over time, is needed in order to say with confidence that one population is affecting the dynamics of another. With a few exceptions [4, 5], there is still a relative paucity of longitudinal studies of appropriate sampling frequency and duration to allow for this approach, and until recently, it has been difficult to apply basic ecological theory to complex microbial communities.

Two of the most influential concepts in modern ecology are foundation and keystone species. As originally defined, a foundation species is ‘a single species that defines much of the structure of a community by creating locally stable conditions for other species, and by modulating and stabilizing fundamental ecosystem processes’ [6]. Further, foundation species are numerically dominant and form close biotic interactions with other community members [7]. Keystone species are similar to foundation species in that they are critical for maintaining the organization and diversity of their ecological communities through biotic interactions with other community members [8]. However, a keystone species is per definition of relatively low abundance, and thus represents a vulnerable point in an ecosystem, the removal of which has strong destabilizing effects, resulting in loss of biodiversity [9, 10]. More diverse communities have enhanced ecosystem function, stability and resistance to invasion [11], and identification of keystone and foundation taxa in the GI microbiota is important as they are potential entrance points for novel diagnostic strategies and therapeutic modulation.

A recently developed method for estimating biotic interactions in complex microbial communities, dubbed reverse ecology (RE) [12], uses genome sequences of species pairs in order to compute indices of metabolic overlap (i.e. exogenous compounds utilized by both species) and complementarity (i.e. compounds produced endogenously by one species which can be utilized by the other). These indices are taken as proxies for competition and facilitation, respectively. A major premise of RE is the concept of limiting similarity. This hypothesis states that competition is stronger between more closely related organisms, as proposed by Darwin in On the Origin of Species [13].

Here, we analyse published data from two longitudinal studies of the GI microbiota that used cutting-edge metagenomics methods [4, 5]. These studies are unique in that they are of long duration (185–443 days) and of sufficient temporal resolution for statistical time series analysis. We present comprehensive mapping of biotic interactions of the GI microbiota in four healthy adults, and we identify putative foundation and keystone taxa. We then compare these results to those obtained with an RE approach and evaluate the limiting similarity hypothesis in this context. Finally, we examine the data through the lens of enterotyping [14], demonstrating both temporal stability and instability within individuals.

## Results

### Mapping biotic interactions

**Fig. 1:**
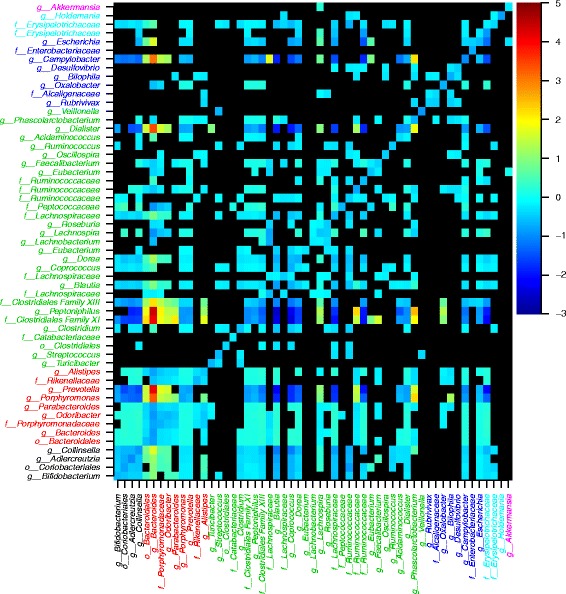
Genus level bacterial interactions for I1. The heat map describes the strength and direction (*β*_*i,j*_ in Eq. 1) of highly significant interactions. Dependent variables are along the *y* axis and independent variables along the *x* axis, i.e. if you follow the column of given genus upward from the *x* axis until you reach a coloured cell, that cell indicates the effect of the given genus (dependent) on the genus indicated on the *y* axis (independent). The *colour key* on the right-hand side indicates the sign and magnitude of interactions that were significant at the 99 % confidence level. Cells representing non-significant relationships are *black*. Genus names are coloured according to phylum provenance: *black Actinobacteria*, *red Bacteroidetes*, *green Firmicutes*, *blue Proteobacteria*, *light blue Tenericutes*, and *pink Verrucomicrobia*. Note that according to the NCBI taxonomy database, the family *Erysipelotrichaceae* and the genus *Holdemania* are classified as *Firmicutes*. Here, and throughout, we have remained consistent with the Greengenes classification of these taxa as *Tenericutes*

Summary statistics of the data used for time series modelling are presented in Table 1. The analysis identified many genus level interactions (Fig. 1, Additional file 1: Figures S1–S7), with considerable variation among subjects (Table 2). Figures presented in the main text are of I1 while I2–4 are discussed in the text, and the corresponding figures are presented in supplementary information. The distributions of the number of data points used to compute the models for each subject are presented in Additional file 1: Figure S8. No relationship was observed between the number of data points used to compute a model and the chance of observing a significant relationship at the 99 % confidence level (Additional file 1: Table S1). When reducing the number of data points used to compute the models, the results were qualitatively very similar to the full models (Table 2), with correlations between regression coefficients at 0.9 or more when reducing the amount of data by 50 % (Additional file 1: Figure S9). However, for the same models, the number of observed significant interactions relative to the total number of potential interactions declined in a near linear fashion as the amount of data was reduced (Additional file 1: Figure S10). In this case, slopes among individuals were relatively consistent with the chance of discovering a significant interaction reduced by 0.6–0.9 % when reducing the data amount by 10 %.

**Table 1:** Summary statistics of data analysed in this study

Individual	Gender	Avg. no. of reads	Duration (days)	No. of samples	No. of genera	Reference
1	Male	21,911	443	332	69	[4]
2	Female	27,461	185	130	48	[4]
3	Male	20,593	365	307	136	[5]
4	Male	20,610	253	181	156	[5]

**Table 2:** Summary of time series modelling results

Individual	Max. no. of data points in model	No. of significant interactions	Intra-specific	Ratio of negative to positive interactions	% significant of total possible interactions
1	269	995	55	2.24	32.9
2	113	157	36	6.85	10.9
3	266	3021	128	3.23	18.4
4	150	2150	120	6.99	12.5

**Fig. 2:**
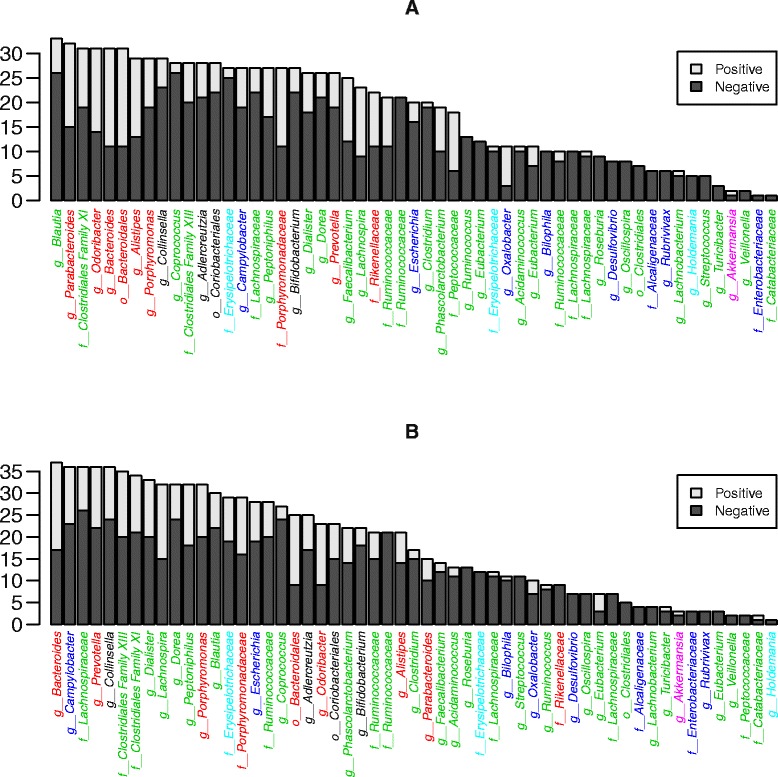
Ranked genus level degree of connectedness in I1. The *bar charts* show the total number of significant negative and positive genus level interactions. Dependent (acted upon) interactions are shown in **a** while independent (acting on) interactions are in **b**. Genus names are coloured according to phylum provenance: *black Actinobacteria*, *red Bacteroidetes*, green *Firmicutes*, *blue Proteobacteria*, *light blue* *Tenericutes* and *pink Verrucomicrobia*

**Fig. 3:**
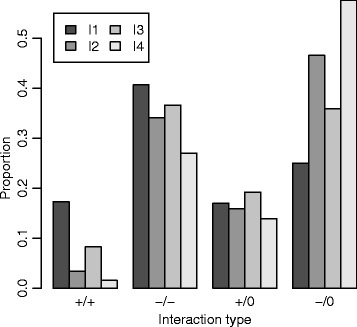
Prevalence of observed pairwise interaction categories. The categories are indicated on the *x* axis: cooperation (+/+), competition (−/−), commensalism (+/0, with zero being a non-significant interaction) and amensalism (−/0). The *y* axis indicates the number of significant interactions in the specified categories (Additional file 1: Figure S11) relative to the total number of pairwise interactions identified in an individual (i.e. the number of pairwise interactions in which at least one taxon in a given pair interacts with the other)

In line with general expectations [15], there was a predominance of negative interactions (Figs. 1 and 2; Table 2; Additional file 1: Figures S1–S7 and S13–S15). I1 and I3 had more positive interactions relative to I2 and I4 (Table 2). We determined the prevalence of pairwise interactions of varying signs in order to assess the frequencies of apparent cooperation (+/+), competition (−/−), exploitation (+/−), commensalism (+/0), with zero being a non-significant interaction and amensalism (−/0). Although there was considerable variability between individuals, pairwise interactions were dominated by competitive and amensal relationships (Fig. 3, Additional file 1: Figure S11). We did not observe a single instance of exploitation in any of the individuals. Overall, biotic interactions were more negative among members of a phylum than between members of different phyla when intra-genus interactions were included in the analysis (*p* < 0.001 for I1–I4). When intra-genus interactions were excluded, the test remained significant for I1, I3 and I4 (*p* < 0.001) but was only marginally significant for I2 (*p* = 0.06).

### Variability in ecological network connectedness

The biotic interactions described above were categorized as positive or negative (i.e. the *β*s in 1 were larger or smaller than zero) and whether they represent a taxa acting upon other taxa (independent variable, right hand term in Eq. 1) or taxa being acted upon (dependent variable, left hand term in Eq. 1). These categories are referred to as positive independent (PI), positive dependent (PD), negative independent (NI) and negative dependent (ND). The sum of all four categories thus represents the degree of connectedness of a taxon.

We observe a large variation in the degree of connectedness across the different genera (Fig. 2, Additional file 1: Figures S13–S15). We examined whether the ratio between positive and negative interactions in which a genus is involved is independent of the genus’ total degree of connectedness and found that more connected genera had disproportionately large numbers of positive interactions relative to less-connected genera in I1 and I3 (*p* < <0.001 for both, linear model), but not I2 and I4 (*p* = 0.845 and 0.974, respectively).

### Bacteriodetes as a candidate foundation taxon

**Fig. 4:**
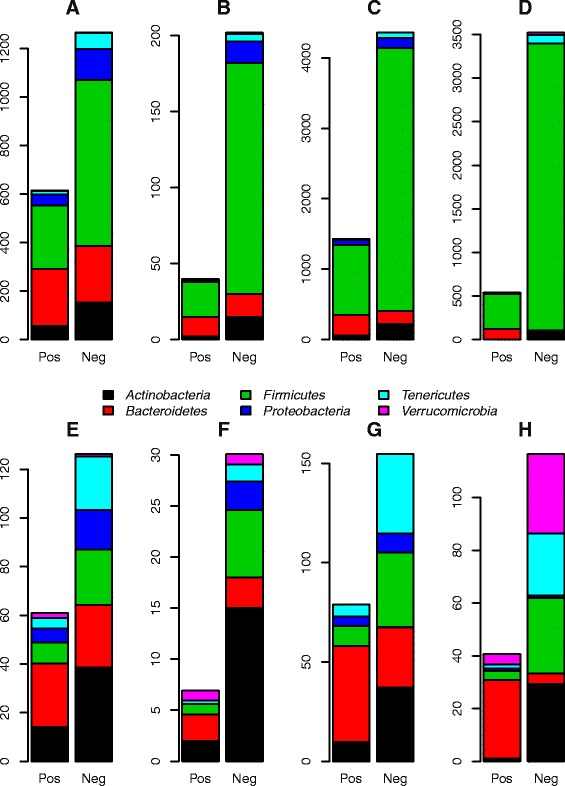
Total and mean connectedness of six main phyla in I1-4. **a**–**d** show counts of positive and negative interactions significant at the 99 % confidence level for I1-4, respectively. **e**–**h** show the mean connectedness of the phyla, i.e. the counts in **a**–**d** divided by the number of observed genera within the respective phyla in each individual. Each bar combines interactions viewed both as dependent and independent. Dependent and independent interactions can be seen separately in Additional file 1: Figure S16

The degree of connectedness in an ecological network can be seen as a proxy for a taxon’s importance in structuring the dynamics of the ecosystem, i.e. its influentialness. *Bacteroidetes* is an abundant taxon (48.8 % mean relative abundance) that is highly connected to the other ecosystem members, in particular through positive interactions (Fig. 2, Additional file 1: Figures S13–S15). When we normalize the degree of connectedness to the number of genera in each phylum, *Bacteroidetes* emerge as the main contributors of positive interactions (Fig. 4, Additional file 1: Figure S16). This observation is supported by linear models testing for differences in the mean number of positive interactions between the different phyla in three of the four individuals (I1, *p* = 0.025; I3, *p* < 0.001; I4, *p* < 0.001). For I2, even though *Bacteroidetes* had the highest mean number of positive interactions, the low total number of positive interactions identified in this individual limits the statistical power to detect significant differences. Thus, the *Bacteroidetes* fit the description of a foundation taxon sensu [6].

### Actinobacteria as a candidate keystone taxon

**Fig. 5:**
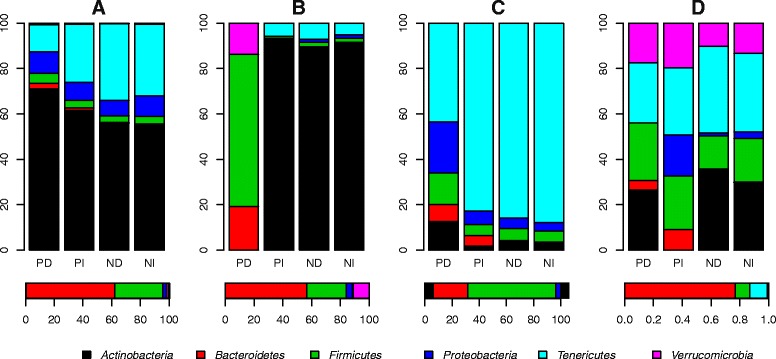
Phylum level degree of connectedness divided by relative abundances. The *bars* show the number of positive dependent (PD), positive independent (PI), negative dependent (ND) and negative independent (NI) interactions observed within each of the six main phyla divided by the mean relative abundance of each phylum (horizontal bars below the panels). The ratios have been converted to percentages. **a**–**d** show data for I1-4, respectively

*Actinobacteria* constitute on average 1.8 % of the GI microbiotas of the four individuals, comprising comparatively few genera. When the degree of connectedness is normalized to relative abundances, it becomes apparent that this group is very influential in the community even though it is relatively scarce (Fig. 5). This pattern is particularly striking in I1, I2 and I4. I3 has a much higher mean abundance of *Actinobacteria* (6.1 %), making it less apparent. The central role of the *Actinobacteria* in the biotic interaction networks, due to large number of both negative and positive interactions, suggests that it may constitute a keystone taxon. This contrasts with the *Bacteroidetes* that are characterized both by high relative abundances and many positive interactions.

**Fig. 6:**
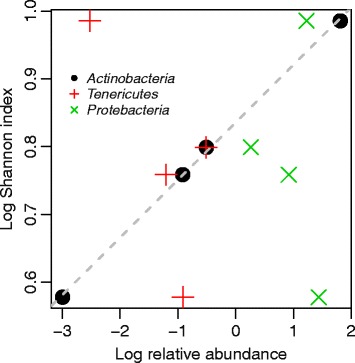
Between-individual relationship between *Actinobacteria* relative abundance and total community diversity (Shannon index). On the log scale, the relationship is linear (*R* ^2^ = 0.99, *p* << 0.001). This relationship was not observed for *Tenericutes* or *Proteobacteria*

Relative abundances of *Actinobacteria* are strongly linked to community beta diversity when viewed across individuals (*R*^2^ = 0.99, *p* << 0.001; Fig. 6). *Proteobacteria* and *Tenericutes* occur at abundances comparable to those of *Actinobacteria*, but no such relationship was observed for these groups. Within individuals, we also observe that variation in diversity is linked with *Actinobacterial* abundances (*p* << 0.001 for all four individuals; Additional file 1: Figure S17). These results further strengthen the position of *Actinobacteria* as a keystone taxon.

### Concordance with interaction maps from a RE approach

The RE approach [12, 16], although fundamentally different from the time series analysis approach taken here, produces results that are qualitatively similar, i.e., they estimate biotic interactions that can be categorized in the same way as our results into PI, PD, NI and ND. Comparison finds concordance between our results, and RE results in that more negative than positive interactions were observed (Additional file 1: Figure S16 E, J) and that more competition was observed within than between groups (*p* << 0.001; Additional file 1: Figure S18). This follows the general expectation that stronger competition would stem from a higher degree of metabolic overlap, which is linked with phylogenetic relatedness.

We also used the complementary and competitive indices as proxies for ecological connectivity (higher value = more connected), summing the indices of species within each of the six main phyla for comparison with time series analysis results (Fig. 4, Additional file 1: Figure S16). The results from RE are similar to the results from time series analysis in that *Firmicutes* and *Bacteroidetes* are the most highly connected groups (Additional file 1: Figure S16A–E). However, in contrast to time series analysis results, the six phyla are roughly equal in terms of mean connectedness estimated from the RE approach (Additional file 1: Figure S16 J).

### Comparison of co-occurrence modelling and time series analysis

Co-occurrence modelling, sometimes used in cross-sectional community studies for inference of biotic interactions, is based on the rationale that negatively and positively correlated occurrence patterns arise from negative and positive interactions, respectively [17]. Here, we analysed co-occurrence of taxa within each of the individuals and compared coefficients of contemporaneous correlation to regression coefficients from time series analysis. In each individual, we found a negative correlation (−0.68, −0.48, −0.70 and −025 for I1–I4, respectively; Spearman correlations) between co-occurrence and time series modelling results. A more detailed analysis (generalized additive models) showed these relationships to be highly significant and mostly non-linear (Additional file 1: Figure S12).

### Using the lens of enterotyping to view longitudinal data

**Fig. 7:**
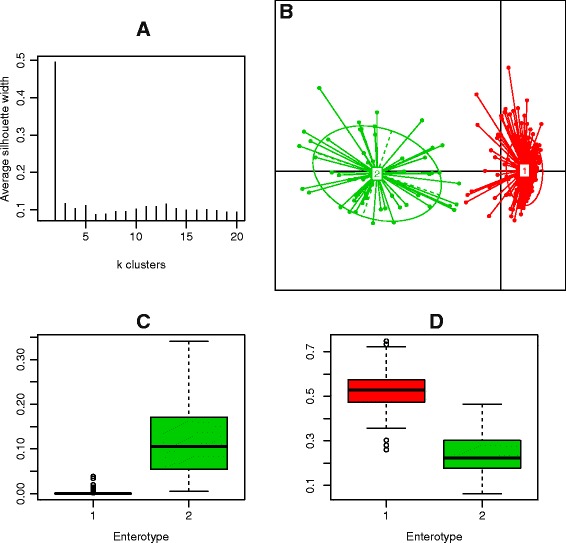
Enterotype switching in I1. **a** Average silhouette widths of different cluster numbers indicate that two clusters provide a reasonable fit to the data. **b** PCoA plot of samples from I1 showing two distinct clusters. **c** Relative abundance of *Prevotella* in samples corresponding to enterotype 1 (*red*) and 2 (*green*). **d** Relative abundance of *Bacteroides* in samples corresponding to enterotype 1 (*red*) and 2 (*green*)

In order to see if enterotypes are stable over time, we looked at temporal patterns of relative abundances of *Bacteriodes*, *Ruminococcus*, and *Prevotella* as well as a species rich fourth group of *Clostridiales* belonging to the *Lachnospiraceae* family and the genus *Blautia* (Additional file 1: Figure S19). These are the main bacterial groups associated with distinct enterotypes in previous publications [14, 18]. For I2, I3 and I4, there was no strong evidence of enterotype clustering (average silhouette widths of 0.23, 0.18 and 0.29, respectively). In addition, for these individuals, there was generally poor agreement between silhouette widths and Calinski-Harabasz indices for determining optimal cluster numbers, further indicating a poor fit to the data. For I1, however, there was support for two distinct clusters (Fig. 7a, b). This is in agreement with previously reported results [19].

Enterotype switching was related to the *Bacteroides/Prevotella* balance (Fig. 7c, d). Enterotype 1, characterized by high *Bacteroides* abundances, was predominant (278 days; 84 %) with type 2 observed for 54 days (16 %). There was some evidence of temporal clustering of enterotype 2 observations (*p* = 0.015, Wald-Wolfowitz test; Additional file 1: Figure S20), but sporadic switching at single time points occurred throughout the time series. Interestingly, in the case of I1, there appears to be a strong dynamic coupling between *Bacteroides* and *Prevotella* (Fig. 1, Additional file 1: Figure S1) with *Bacteroides* exerting a strong positive influence on *Prevotella* and a less pronounced yet still significant positive reciprocal interaction. This relationship was not observed in the three other individuals and may be related to the switching phenotype observed for I1.

## Discussion

### Evaluation of the limiting similarity hypothesis

As species of the same genus have usually, though by no means invariably, some similarity in habits and constitution, and always in structure, the struggle will generally be more severe between species of the same genus, when they come into competition with each other, than between species of different genera [13].

Darwin’s limiting similarity hypothesis has been hotly debated with evidence both in favour of [20–22] and against [23–25]. The results presented here, as well a data from RE modelling, suggest that Darwin might have been right in the case of the GI microbiota. Our results agree with the general assumption of RE that there is a positive relationship between strength of competition and niche overlap, as measured by metabolic similarity inferred from full genome sequences. We can see this from the elevated intensity of negative interactions within relative to between phyla (Fig. 1, Additional file 1: Figures S1–S7 and S18). From RE, it is evident that the degree of within phylum metabolic overlap is generally higher than between members of different phyla, and our results corroborate the assertion that this results in stronger competition.

Levy and Borenstein [16] used co-occurrence patterns obtained from cross-sectional data along with RE to discriminate between habitat filtering and species assortment as community assembly rules for the human GI microbiota, finding that strongly competing species tend to co-occur more often than expected. Our results are in general agreement with the conclusion that habitat filtering is the main assembly rule in the GI microbiota since we found significant negative relationships between co-occurrence and interaction coefficients estimated by time series analysis (Additional file 1: Figure S12), demonstrating that competing taxa also tend to co-occur within individuals.

### Differences between time series analysis and RE

While our results are similar to RE in some respects, there are also some fundamental differences. RE assumes that all community members compete with each other since there is a degree of metabolic and genomic overlap in all bacteria. This leads to perfect connectedness between all community members, making it difficult to identify taxa that are particularly important in structuring the community. Our approach found that most genera only interact with a few others (Table 2). This could be the result of stratification within the gastrointestinal tract [26], sequestering sub-populations of bacteria into separate regions. RE also limits interactions to known metabolic pathways and will inevitably fail to identify indirect interactions. RE, while undoubtedly producing important insight, estimates the potential for competition and cross-feeding rather than actual biotic interactions, without considering the environment. It is well known that the nature of pairwise biotic interactions is highly dependent on the environmental context [27, 28].

RE may be expected to improve in the future as more genomes and metabolic pathways are characterized. The time series approach, however, being based on observational population data, does not require some of the strong assumptions of RE.

### Ecological roles: foundation and keystone species

Identification of ecological roles of GI community members is an important step towards understanding how stable, diverse and healthy GI microbial ecosystems are maintained. Current approaches to GI microbiota modulation, usually probiotic or prebiotic, are not based on mechanistic concepts, and current evidence has been insufficient to substantiate health benefits. However, these approaches are attractive as agents of intervention against, and prevention of, a number of maladies. In 2013, the global market value for probiotics was estimated at US$32 billion, and this number is expected to grow to US$52 billion by 2020 [29].

The concepts of foundation and keystone species are used in conservation biology in order to identify and focus efforts on taxa that are particularly important in maintaining the structure, diversity and ultimately the function of ecological systems. Taxa fulfilling these criteria could also be considered prime targets for maintenance of intestinal health through manipulation of the GI microbiota. The GI microbiota has been described as a functionally redundant ecosystem [30] that may be less reliant on particular species, and the role of specific taxa as community stabilizers has hitherto not been thoroughly addressed. Here, we expand foundation and keystone species concepts to apply to higher taxa, analogous to guilds or functional groups, in order to better fit the community structures observed in the GI tract. Species level characterization of complex bacterial communities is very difficult. Indeed, the entire species concept for bacteria is subject to much debate [31]. By grouping bacteria that are phylogenetically and functionally closely related into more informative units, we can begin to assign general ecological roles.

In our analyses, the *Bacteroidetes* stood out as an abundant group that is highly connected in the ecological network. In particular, they were involved in many positive interactions, which are known to be important ecosystem stabilizers because of their potential to cascade through the community with major effects on the structure and function [7, 32, 33]. *Bacteriodetes* spp. can break down complex polysaccharides that would otherwise be inaccessible to most other gut-adapted bacteria [33–36], in particular host-derived and plant glycans [37]. Thus, by making additional nutrients available to other community members, *Bacteroidetes* could act as a foundation taxon through facilitation. Indeed, a previous study found species of *Bacteroides* to be highly connected in the ecological network of the GI microbiota [38], and reduced *Bacteroidetes* abundances have been associated with obesity, indicating a fundamental role for these bacteria in maintaining a healthy GI microbiota [39].

The keystone species concept has previously been used in the context of the GI microbiota for describing bacteria that are instrumental to the biodegradation of resistant starch [40]. There is also evidence that certain *Actinobacteria* are particularly adept at degrading this class of carbohydrates [35, 41, 42], suggesting that they could play a similar role. In our analyses, the *Actinobacteria* fit the definition of a keystone taxon since they are characterized by an influentialness in the community that is disproportional to their low relative abundance, due to the high number of biotic interactions in which they are involved. High abundances of *Actinobacteria* are also clearly associated with increased bacterial diversity in the GI (Fig. 6, Additional file 1: Figure S17), although our analyses cannot establish whether this group of bacteria is a cause or a result of this observation. *Actinobacteria* strains have long been used as probiotic agents, in particular *Bifidobacteria* spp. [43]. Inflammatory bowel disease (IBD) patients have been found to be deficient for *Collinsella* spp. [44, 45] suggesting a role for this genus in the prevention of dysbiosis.

### Temporal stability of enterotypes and links to biotic interaction structure

Clustering of a set of GI microbial community profiles according to a distance metric has been used to categorize individual microbiotas in large cross-sectional studies [14] and to link GI microbiota profiles with dietary patterns [18]. Recently, however, the concept has been drawn into question as being overly simplistic, and it has been proposed that GI microbiota differences may be better described by continuous gradients rather than sharply bounded enterotypes [46]. Although controversial, potential benefits from classification of complex microbial communities by projection onto a lower dimensional space, e.g. for diagnostic purposes, mean that enterotyping and similar approaches should be carefully considered. A recent study re-analysing data from I1 found that the enterotype of a single individual can be unstable over time [19]. We observed the same changes in I1 but found no evidence for multiple clusters in the other three individuals examined. From our analyses, there is some indication that enterotype switching could be associated with a strong positive but asymmetric interaction between *Bacteroides* and *Prevotella* observed in I1 (Fig. 7), although it is hard to see why this would make the individual prone to switching. Relationships between community stability and structural properties, including mutualistic interactions, is an enduring problem in ecology [47] and larger longitudinal studies will be needed to further evaluate the preponderance of, as well as mechanisms behind, enterotype switching.

## Conclusions

Although the time series data compiled in the two studies [4, 5] that supplied data for our analyses are impressive in terms of temporal scope and sampling frequency, they produced data for only four individuals. To our knowledge, these are presently the only two studies of this kind, and a sample size of four is rather small for drawing general conclusions. Although we observed large individual differences, both in taxon composition and biotic interaction structure, some of our observations, i.e., limiting similarity and highly connected taxa, are consistent across individuals. Our results are also in broad agreement with RE. Although logistically challenging, large longitudinal studies with many participants will be needed to further validate the results presented here.

Interestingly, although methodologically very similar, the two studies we examine show variability in their results that appears to be systematic. In particular, I3 and I4 [5] were found to have expanded diversity within the *Firmicutes* compared with I1 and I2 [4]. Whether these represent actual differences in community structures or are due to technical differences in sample/data processing is unclear. Our filtering procedures were standardized throughout and we tried to apply conservative cutoffs to reduce the possibility of discovering spurious interactions. It is well known that metagenomic amplicon sequencing can be extremely sensitive to even small variations in protocol, and efforts towards standardization in order to ensure comparability across studies should be a future priority.

## Methods

### Time series data

The data used in this study were operational taxonomic unit (OTU) tables from two longitudinal studies of the healthy adult GI microbiota [4, 5]. Each study investigated two individuals that we subsequently refer to as I1, I2, I3 and I4 (Table 1). In each study, the V4 region of the 16S rRNA gene was sequenced on an Illumina GAIIx using the same protocol [48]. Sequence reads were processed with the QIIME [49] software suite, and 97 % OTUs were picked against the Greengenes database using uclust [50]. Prior to our analysis, the following additional processing steps were applied: (1) Only the sample with the largest library size was kept if multiple samples were taken on a single day. (2) Singletons were removed. (3) Samples with a library size of less than 20,000 reads were removed. (4) A common scaling procedure was applied as recommended by McMurdie and Holmes [51]. This entails multiplying every OTU count in a given library with a factor that is the ratio of the smallest library size in the data set to the library size of the sample in question. This replaces rarefying (i.e. random sub-sampling to the lowest number of reads) as it effectively results in the library scaling one would achieve by averaging an infinite number of repeated sub-samplings. (5) OTU tables were collapsed to the genus level by merging counts of species within the same genus. (6) OTUs with a mean relative abundance of less than 0.01 % were removed in order to reduce noise.

### Reverse ecology data

Competition and complementarity indices were taken from Levy and Borenstein [16], where pairwise competition indices were estimated from genome sequences based on the number of exogenous metabolites utilized by both species. These values were used as proxies for the intensity of competition. This was then normalized to a value between 0 and 1 where 0 means no competition and 1 signifies complete metabolic overlap, i.e. intra-specific competition. Similarly, the complementarity index between two species was estimated as the number of compounds produced by one species that can be used by the second. This served as a proxy for positive interactions. For our analysis, we reduced the table of 154 species to 97 species corresponding to the 6 phyla represented in the time series data.

### Time series modelling

The dynamics of each genus level OTU was modelled according to the function,

1 $$ {x}_{i,t+\mathsf{1}}\hbox{-} {x}_{i,t}={\alpha}_{i,j}+{\beta}_{i,j}{x}_{j,t} $$

where *x*_*i,t*_ is the log relative abundance of taxon *i* at time = *t*, *α*_*i,j*_ are intercept terms, *β*_*i,j*_ are linear regression coefficients and *x*_*j,t*_ are log relative abundances of taxon *j* at time = *t*. If *i* = *j*, the estimated interaction is within a given genus which is expected to be strongly negative due to density-dependent competition. The total number of equations in a system is equal to *n*^2^, where *n* is the total number of taxa. This approach does not capture relationships that are strongly non-linear, that could be modelled, e.g. by generalized additive models, but previous work has shown linear regression to be a good approximation [3]. OTUs observed in fewer than 50 % of samples for a given subject were excluded from regression analysis. Prior to model computation, samples that did not have another sample taken the day directly after it were dropped. Only non-zero data points were used for modelling, and models based on fewer than 50 non-zero points were excluded from further analysis. A 99 % confidence level (*p* ≤ 0.01) was used for a model to be considered significant. For computing models with data amounts reduced relative to the full models (Table 2), data pairs (*x*_*i,t*+1_ and *x*_*j,t*_) were sampled randomly to a series of maximal numbers for each individual before model computation. The maximal numbers of samples for I1 and I3 were 250, 200, 150, 125, 100, 75, 50 and 25. For I2, they were 100, 75, 50 and 25. For I4, they were 125, 100, 75, 50 and 25. For each maximal number of data points, models were computed 100 times for each individual, using the filtering criteria stated above (except in the case of 25 data points where the minimal number was reduced from 50 to 25). In order to assess the correspondence between the full and reduced models, we used two metrics. First, we looked at the correlation between regression coefficients between full and reduced models. The coefficients were found to be roughly normally distributed by visual inspection, so we used the standard Pearson correlation for this metric. We also looked at the number of interactions significant at the 99 % confidence level relative to the total number of potential interactions (square of the interaction matrix dimension) for each individual for full and reduced models in order to assess the power of detection. Both correlations and power of detection values are reported as means of 100 model computations. The interaction matrix diagonals (inter-taxon interactions) were omitted from these analyses. All computations were carried out using R [52].

### Tests for differences in interaction structure within and between groups

Tests were carried out by comparing the regression coefficients (*β*s) of significant models describing interactions within phyla with the coefficients of models describing interactions between members of different phyla. For the full test, this entailed pooling values from all within phylum models and all values from between phylum models. Comparisons were then carried out using Wilcoxon rank-sum tests (a.k.a. Mann-Whitney *U* tests). Since the *β*s from models describing intra-genus interactions were always highly negative, tests were carried out both with and without these values. For the RE data, the same test was carried out on a matrix containing the differences between the complementarity and competition indices, excluding the matrix diagonal elements.

### Comparison of co-occurrence and time series analysis

For determining co-occurrence patterns, we first computed pairwise contemporaneous Spearman correlation coefficients between the different taxa within each individual. In order to ensure that the taxa included in the analysis were the same as those included in the time series models, OTUs observed in fewer than 50 % of samples for a given subject were excluded from the analyses. Since the distribution correlation coefficients from an individual sometimes deviated from normality, Spearman correlation was again employed in order to assess the relationship with regression coefficients obtained by time series regression analyses. This relationship was also assessed using generalize additive models as implemented in the R package ‘mgcv’, using 3 degrees of freedom in order to accommodate non-linear relationships.

### Enterotyping

Enterotyping was carried out according to Arumugam et al. [14] using the square root of Jensen-Shannon divergences as a distance metric. Abundance profiles were clustered using partitioning around medioids as implemented in the R-package ‘cluster’. Optimal cluster numbers were determined using both the Calinski-Harabasz index [53] implemented in the ‘clusterSim’ R-package and silhouette widths [54] in the ‘cluster’ package. Principal coordinates analysis (PCoA) and visualization of results were carried out using the ‘ade4’ R-package. Testing for temporal enterotype clustering was done using the Wald-Wolfowitz test for randomness of the distribution of values in a vector [55] implemented in the R-package ‘adehabitatLT’.

### Availability of supporting data

Supplementary information is included with the article and available on the Microbiome website.

## Additional file

Additional file 1:
**The additional file accompanying this article contains Figures S1–20 and Table S1.** (DOCX 753 kb)

## Abbreviations

GI: gastrointestinal
IBD: inflammatory bowel disease
ND: negative dependent
NI: negative independent
OTU: operational taxonomic unit
PCoA: principal coordinates analysis
PD: positive dependent
PI: positive independent
RE: reverse ecology

## References

[1] Sommer F, Backhed F (2013). The gut microbiota—masters of host development and physiology. Nat Rev Microbiol.

[2] Greenblum S, Chiu HC, Levy R, Carr R, Borenstein E (2013). Towards a predictive systems-level model of the human microbiome: progress, challenges, and opportunities. Curr Opin Biotechnol.

[3] Trosvik P, de Muinck EJ, Stenseth NC (2015). Biotic interactions and temporal dynamics of the human gastrointestinal microbiota. ISME J.

[4] Caporaso JG, Lauber CL, Costello EK, Berg-Lyons D, Gonzalez A, Stombaugh J (2011). Moving pictures of the human microbiome. Genome Biol.

[5] David LA, Materna AC, Friedman J, Campos-Baptista MI, Blackburn MC, Perrotta A (2014). Host lifestyle affects human microbiota on daily timescales. Genome Biol.

[6] Dayton PK, Parker BC (1972). Toward and understanding of community resilience and the potential effects of enrichment to the benthos at McMurdo Sound, Antarctica. Proceedings of the colloquium on conservation problems in antarctica.

[7] Stachowicz JJ (2001). Mutualism, facilitation, and the structure of ecological communities. Bioscience.

[8] Paine RT (1969). A note on trophic complexity and community stability. Am Nat.

[9] Stachowicz JJ, Hay ME (1999). Mutualism and coral persistence. The role of herbivore resistance to algal chemical defense. Ecology.

[10] Witman JD (1987). Subtidal coexistence—storms, grazing, mutualism, and the zonation of kelps and mussels. Ecol Monogr.

[11] Chapin FS, Sala OE, Burke IC, Grime JP, Hooper DU, Lauenroth WK (1998). Ecosystem consequences of changing biodiversity—experimental evidence and a research agenda for the future. Bioscience.

[12] Levy R, Borenstein E (2012). Reverse ecology: from systems to environments and back. Adv Exp Med Biol.

[13] Darwin C (1859). On the origin of species by means of natural selection.

[14] Arumugam M, Raes J, Pelletier E, Le Paslier D, Yamada T, Mende DR (2011). Enterotypes of the human gut microbiome. Nature.

[15] Foster KR, Bell T (2012). Competition, not cooperation, dominates interactions among culturable microbial species. Current Bio.

[16] Levy R, Borenstein E (2013). Metabolic modeling of species interaction in the human microbiome elucidates community-level assembly rules. Proc Natl Acad Sci U S A.

[17] Faust K, Raes J (2012). Microbial interactions: from networks to models. Nat Rev Microbiol.

[18] Wu GD, Chen J, Hoffmann C, Bittinger K, Chen YY, Keilbaugh SA (2011). Linking long-term dietary patterns with gut microbial enterotypes. Science.

[19] Knights D, Ward TL, McKinlay CE, Miller H, Gonzalez A, McDonald D, Knight R (2014). Rethinking “enterotypes”. Cell Host Microbe.

[20] Burns JH, Strauss SY (2012). More closely related species are more ecologically similar in an experimental test (vol 108, pg 5302, 2011). Proc Natl Acad Sci U S A.

[21] Jiang L, Tan JQ, Pu ZC (2010). An experimental test of Darwin’s naturalization hypothesis. Am Nat.

[22] Violle C, Nemergut DR, Pu ZC, Jiang L (2011). Phylogenetic limiting similarity and competitive exclusion. Ecol Lett.

[23] Cahill JF, Kembel SW, Lamb EG, Keddy PA (2008). Does phylogenetic relatedness influence the strength of competition among vascular plants?. Perspect Plant Ecol.

[24] Fritschie KJ, Cardinale BJ, Alexandrou MA, Oakley TH (2014). Evolutionary history and the strength of species interactions: testing the phylogenetic limiting similarity hypothesis. Ecology.

[25] Godoy O, Kraft NJB, Levine JM (2014). Phylogenetic relatedness and the determinants of competitive outcomes. Ecol Lett.

[26] Stearns JC, Lynch MDJ, Senadheera DB, Tenenbaum HC, Goldberg MB, Cvitkovitch DG, et al. Bacterial biogeography of the human digestive tract. Sci Rep-Uk. 2011;1. 10.1038/srep00170 PMC3240969 22355685

[27] Bronstein JL (1994). Conditional outcomes in mutualistic interactions. Trends Ecol Evol.

[28] de Muinck EJ, Stenseth NC, Sachse D, Vander Roost J, Ronningen KS, Rudi K, Trosvik P (2013). Context-dependent competition in a model gut bacterial community. PLoS One.

[29] Grand View Research (2014). Probiotics market analysis by application (probiotic functional foods & beverages, probiotic dietary supplements, animal feed probiotics).

[30] Lozupone CA, Stombaugh JI, Gordon JI, Jansson JK, Knight R (2012). Diversity, stability and resilience of the human gut microbiota. Nature.

[31] Cohan FM (2002). What are bacterial species?. Annu Rev Microbiol.

[32] Callaway RM, Brooker RW, Choler P, Kikvidze Z, Lortie CJ, Michalet R (2002). Positive interactions among alpine plants increase with stress. Nature.

[33] Cavender-Bares J, Kozak KH, Fine PVA, Kembel SW (2009). The merging of community ecology and phylogenetic biology. Ecol Lett.

[34] Lee SM, Donaldson GP, Mikulski Z, Boyajian S, Ley K, Mazmanian SK (2013). Bacterial colonization factors control specificity and stability of the gut microbiota. Nature.

[35] Macfarlane GT, Englyst HN (1986). Starch utilization by the human large intestinal microflora. J Appl Bacteriol.

[36] Xu J, Bjursell MK, Himrod J, Deng S, Carmichael LK, Chiang HC (2003). A genomic view of the human—*Bacteroides thetaiotaomicron* symbiosis. Science.

[37] Martens EC, Chiang HC, Gordon JI (2008). Mucosal glycan foraging enhances fitness and transmission of a saccharolytic human gut bacterial symbiont. Cell Host Microbe.

[38] Fisher CK, Mehta P (2014). Identifying keystone species in the human gut microbiome from metagenomic timeseries using sparse linear regression. PLoS One.

[39] Ley RE, Turnbaugh PJ, Klein S, Gordon JI (2006). Microbial ecology—human gut microbes associated with obesity. Nature.

[40] Ze XL, Duncan SH, Louis P, Flint HJ (2012). *Ruminococcus bromii* is a keystone species for the degradation of resistant starch in the human colon. Isme J.

[41] Ryan SM, Fitzgerald GF, van Sinderen D (2006). Screening for and identification of starch-, amylopectin-, and pullulan-degrading activities in bifidobacterial strains. Appl Environ Microbiol.

[42] Salyers AA, West SE, Vercellotti JR, Wilkins TD (1977). Fermentation of mucins and plant polysaccharides by anaerobic bacteria from the human colon. Appl Environ Microbiol.

[43] Nicholson JK, Holmes E, Kinross J, Burcelin R, Gibson G, Jia W, Pettersson S (2012). Host-gut microbiota metabolic interactions. Science.

[44] Kassinen A, Krogius-Kurikka L, Makivuokko H, Rinttila T, Paulin L, Corander J (2007). The fecal microbiota of irritable bowel syndrome patients differs significantly from that of healthy subjects. Gastroenterology.

[45] Lyra A, Rinttila T, Nikkila J, Krogius-Kurikka L, Kajander K, Malinen E (2009). Diarrhoea-predominant irritable bowel syndrome distinguishable by 16S rRNA gene phylotype quantification. World J Gastroentero.

[46] Jeffery IB, Claesson MJ, O’Toole PW, Shanahan F (2012). Categorization of the gut microbiota: enterotypes or gradients?. Nat Rev Microbiol.

[47] Okuyama T, Holland JN (2008). Network structural properties mediate the stability of mutualistic communities. Ecol Lett.

[48] Caporaso JG, Lauber CL, Walters WA, Berg-Lyons D, Lozupone CA, Turnbaugh PJ (2011). Global patterns of 16S rRNA diversity at a depth of millions of sequences per sample. Proc Natl Acad Sci U S A.

[49] Caporaso JG, Kuczynski J, Stombaugh J, Bittinger K, Bushman FD, Costello EK (2010). QIIME allows analysis of high-throughput community sequencing data. Nat Methods.

[50] Edgar RC (2010). Search and clustering orders of magnitude faster than BLAST. Bioinformatics.

[51] McMurdie PJ, Holmes S (2014). Waste not, want not: why rarefying microbiome data is inadmissible. PLoS Comput Biol.

[52] R Core Team (2014). R: A language and environment for statistical computing.

[53] Calinski TH, Harabasz J (1974). A dendrite method for cluster analysis. Comput Stat.

[54] Rousseeuw PJ (1987). Silhouettes: a graphical aid to the interpretation and validation of cluster analysis. J Comput Appl Math.

[55] Wald A, Wolfowitz J (1943). An exact test for randomness in the non-parametric case based on serial correlation. Ann Math Stat.

