# Supplementary material for: Ecology of bacteria in the human gastrointestinal tract—identification of keystone and foundation taxa
Source: Microbiome. 2015 Oct 12;3:44. doi: 10.1186/s40168-015-0107-4 (PMC4601151; doi:10.1186/s40168-015-0107-4)
Supplement: Additional file 1: — The additional file accompanying this article contains Figures S1–20 and Table S1. (DOCX 753 kb) [file 40168_2015_107_MOESM1_ESM.docx]

Additional file 1: Table S1, Figures S1-S20

**Table S1**

|  | A | B | C | D |
| --- | --- | --- | --- | --- |
| Significant | 195 | 101 | 251 | 102 |
| Non-significant | 197 | 93 | 236 | 100 |

Median nr. of data points used for computing that were found to be significant and non-significant, respectively.

**
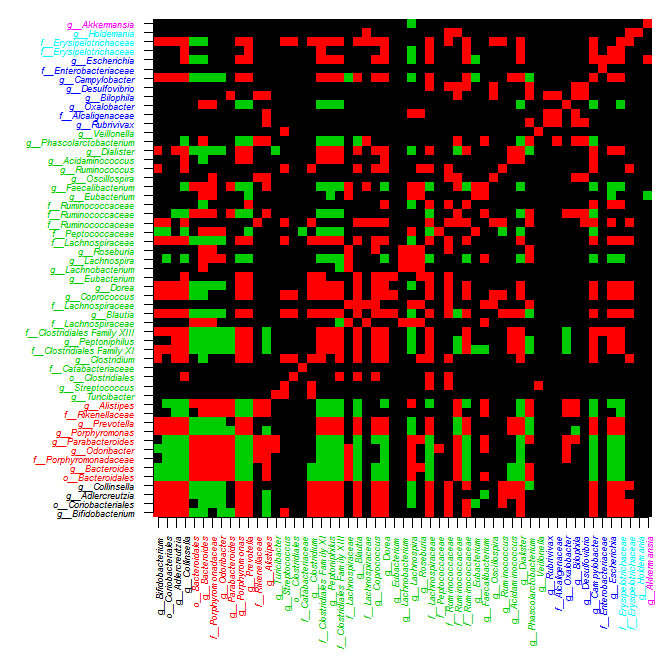
**

**Figure S1. Sign of significant genus level bacterial interactions for I1.** The plot describes the direction (β_i,j_ in Eq.1) of highly significant interactions. Dependent variables are along the y axis and independent variables along the x axis, i.e. if you follow the column of given genus upward from the x-axis until you reach a coloured cell that cell indicates the effect of the given genus (dependent) on the genus indicated on the y-axis (independent). The colour indicates the sign (green=positive, red=negative) of interactions that were significant at the 99% confidence level. Cells representing non-significant relationships are black. Genus names are coloured according to phylum provenance: Black= *Actinobacteria*, Red= *Bacteroidetes*, Green= *Firmicutes*, Blue=*Proteobacteria*, Light Blue=*Tenericutes*, and Pink=*Verrucomicrobia*

**
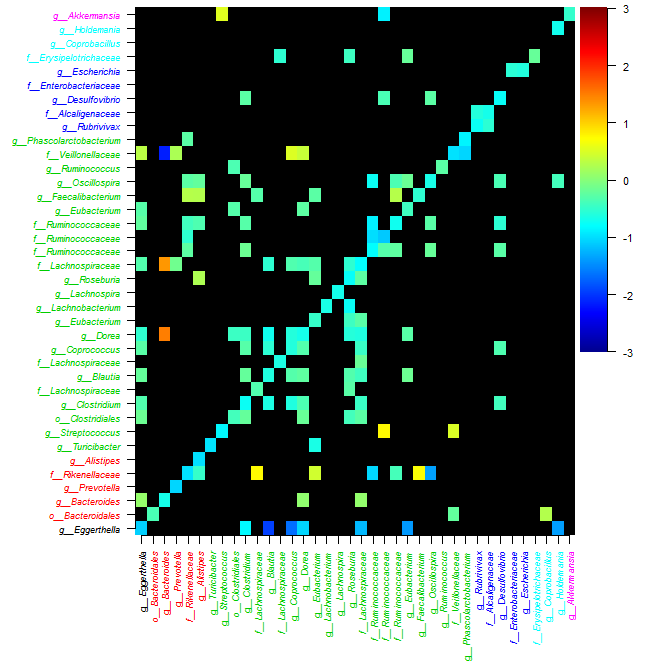
Figure S2. Genus level bacterial interactions for I2.** The heat map describes the strength and direction (β_i,j_ in Eq.1) of highly significant interactions. Dependent variables are along the y axis and independent variables along the x axis, i.e. if you follow the column of given genus upward from the x-axis until you reach a coloured cell that cell indicates the effect of the given genus (dependent) on the genus indicated on the y-axis (independent). The colour key on the right-hand side indicates the sign and magnitude of interactions that were significant at the 99% confidence level. Cells representing non-significant relationships are black. Genus names are coloured according to phylum provenance: Black= *Actinobacteria*, Red= *Bacteroidetes*, Green= *Firmicutes*, Blue=*Proteobacteria*, Light Blue=*Tenericutes*, and Pink=*Verrucomicrobia*

**
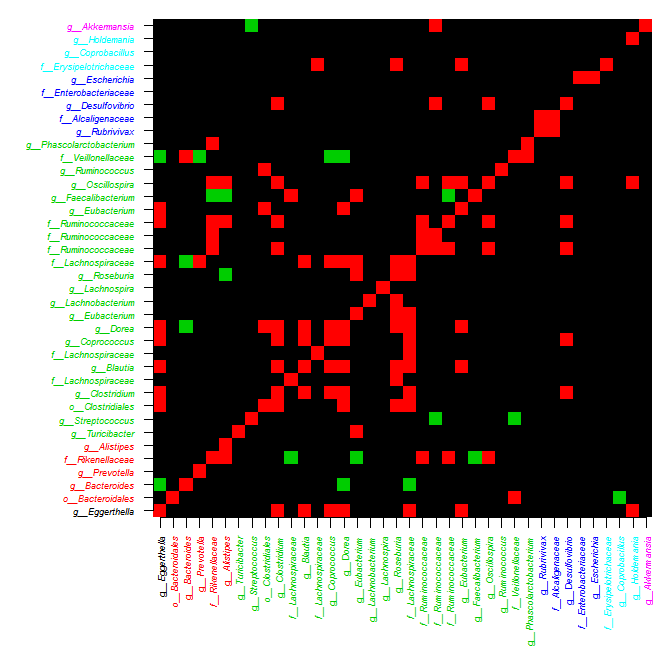
Figure S3. Sign of significant genus level bacterial interactions for I2.** The plot describes the direction (β_i,j_ in Eq.1) of highly significant interactions. Dependent variables are along the y axis and independent variables along the x axis, i.e. if you follow the column of given genus upward from the x-axis until you reach a coloured cell that cell indicates the effect of the given genus (dependent) on the genus indicated on the y-axis (independent). The colour indicates the sign (green=positive, red=negative) of interactions that were significant at the 99% confidence level. Cells representing non-significant relationships are black. Genus names are coloured according to phylum provenance: Black= *Actinobacteria*, Red= *Bacteroidetes*, Green= *Firmicutes*, Blue=*Proteobacteria*, Light Blue=*Tenericutes*, and Pink=*Verrucomicrobia*

**
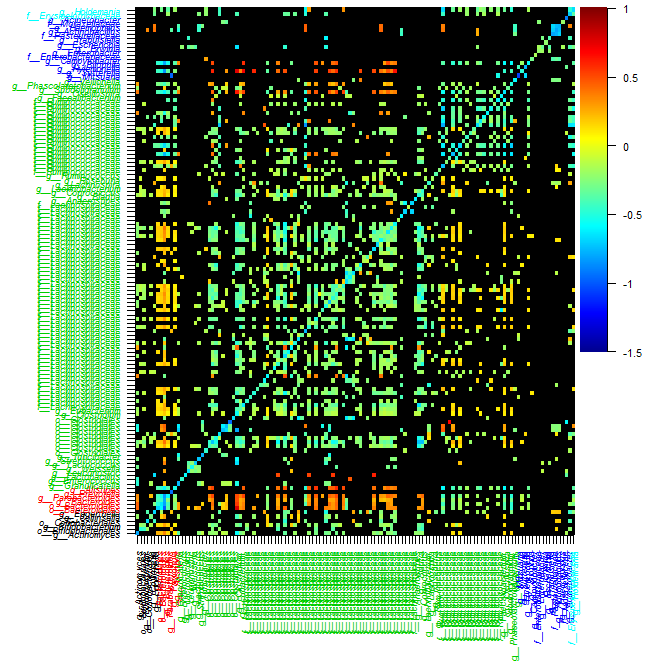
Figure S4. Genus level bacterial interactions for I3.** The heat map describes the strength and direction (β_i,j_ in Eq.1) of highly significant interactions. Dependent variables are along the y axis and independent variables along the x axis, i.e. if you follow the column of given genus upward from the x-axis until you reach a coloured cell that cell indicates the effect of the given genus (dependent) on the genus indicated on the y-axis (independent). The colour key on the right-hand side indicates the sign and magnitude of interactions that were significant at the 99% confidence level. Cells representing non-significant relationships are black. Genus names are coloured according to phylum provenance: Black= *Actinobacteria*, Red= *Bacteroidetes*, Green= *Firmicutes*, Blue=*Proteobacteria*, Light Blue=*Tenericutes*, and Pink=*Verrucomicrobia*

**
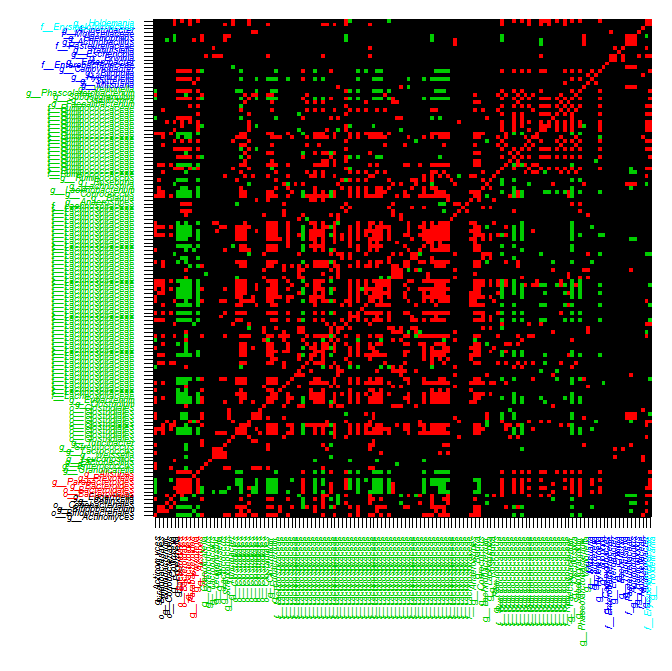
Figure S5. Sign of significant genus level bacterial interactions for I3.** The plot describes the direction (β_i,j_ in Eq.1) of highly significant interactions. Dependent variables are along the y axis and independent variables along the x axis, i.e. if you follow the column of given genus upward from the x-axis until you reach a coloured cell that cell indicates the effect of the given genus (dependent) on the genus indicated on the y-axis (independent). The colour indicates the sign (green=positive, red=negative) of interactions that were significant at the 99% confidence level. Cells representing non-significant relationships are black. Genus names are coloured according to phylum provenance: Black= *Actinobacteria*, Red= *Bacteroidetes*, Green= *Firmicutes*, Blue=*Proteobacteria*, Light Blue=*Tenericutes*, and Pink=*Verrucomicrobia*

**
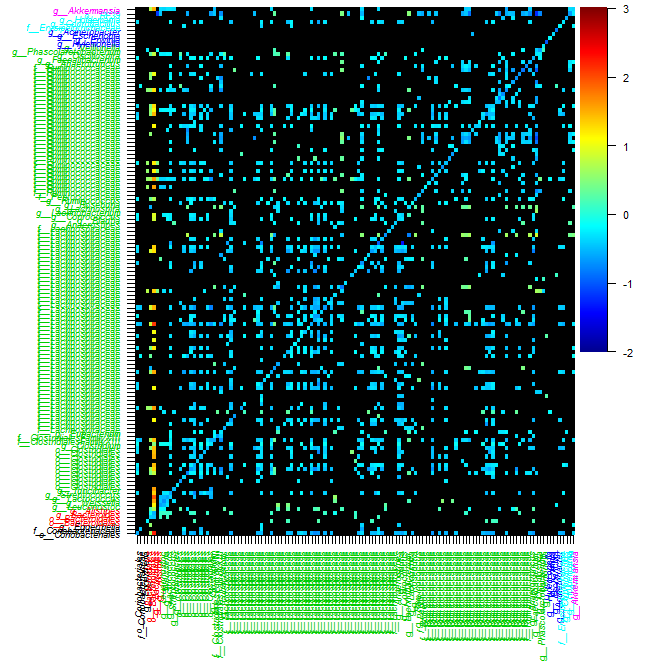
Figure S6. Genus level bacterial interactions for I4.** The heat map describes the strength and direction (β_i,j_ in Eq.1) of highly significant interactions. Dependent variables are along the y axis and independent variables along the x axis, i.e. if you follow the column of given genus upward from the x-axis until you reach a coloured cell that cell indicates the effect of the given genus (dependent) on the genus indicated on the y-axis (independent). The colour key on the right-hand side indicates the sign and magnitude of interactions that were significant at the 99% confidence level. Cells representing non-significant relationships are black. Genus names are coloured according to phylum provenance: Black= *Actinobacteria*, Red= *Bacteroidetes*, Green= *Firmicutes*, Blue=*Proteobacteria*, Light Blue=*Tenericutes*, and Pink=*Verrucomicrobia*

**
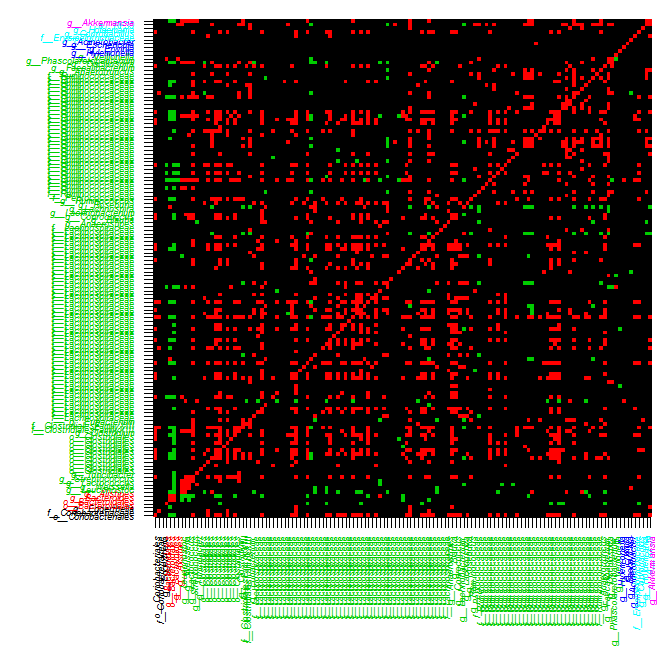
Figure S7. Sign of significant genus level bacterial interactions for I4.** The plot describes the direction (β_i,j_ in Eq.1) of highly significant interactions. Dependent variables are along the y axis and independent variables along the x axis, i.e. if you follow the column of given genus upward from the x-axis until you reach a coloured cell that cell indicates the effect of the given genus (dependent) on the genus indicated on the y-axis (independent). The colour indicates the sign (green=positive, red=negative) of interactions that were significant at the 99% confidence level. Cells representing non-significant relationships are black. Genus names are coloured according to phylum provenance: Black= *Actinobacteria*, Red= *Bacteroidetes*, Green= *Firmicutes*, Blue=*Proteobacteria*, Light Blue=*Tenericutes*, and Pink=*Verrucomicrobia*

**
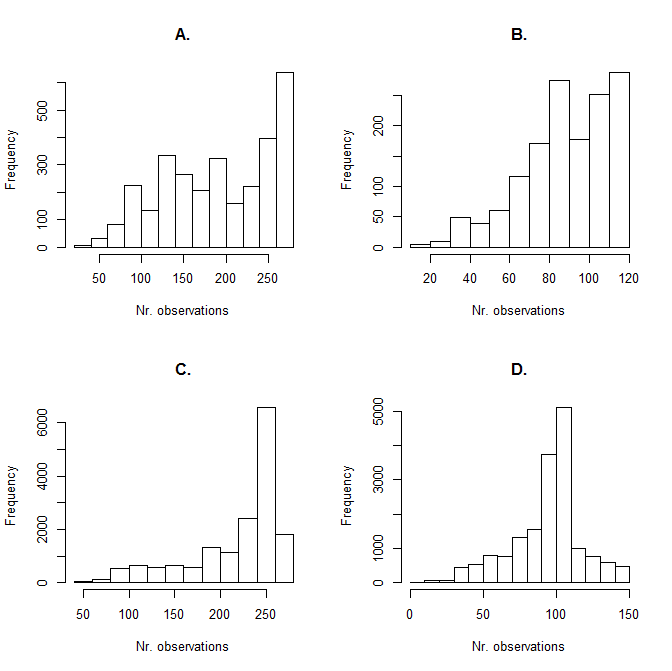
**

**Figure S8.** Histograms of nr. data points used for computing time series regression models for I1 (A), I2 (B), I3 (C) and I4 (D). Models based on fewer than 50 paired observations were excluded from further analysis.


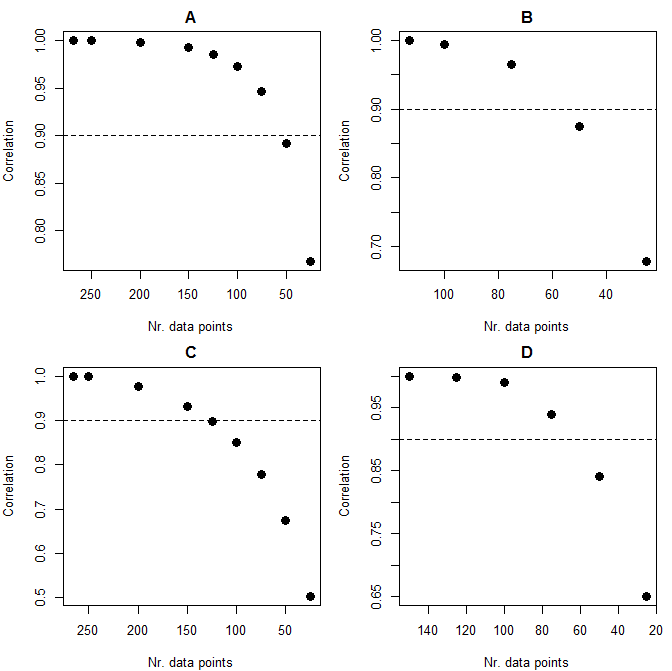


**Figure S9.** Effects of the number of data points used to estimate time series regression models. The y-axes show the correlation between regression coefficients computed using the full data sets and models using the number of data points specified on the x-axes. The full models (Table 2) have been included in the graphs for interpretability (leftmost points). Panels A-D represent results for I1-I4, respectively.


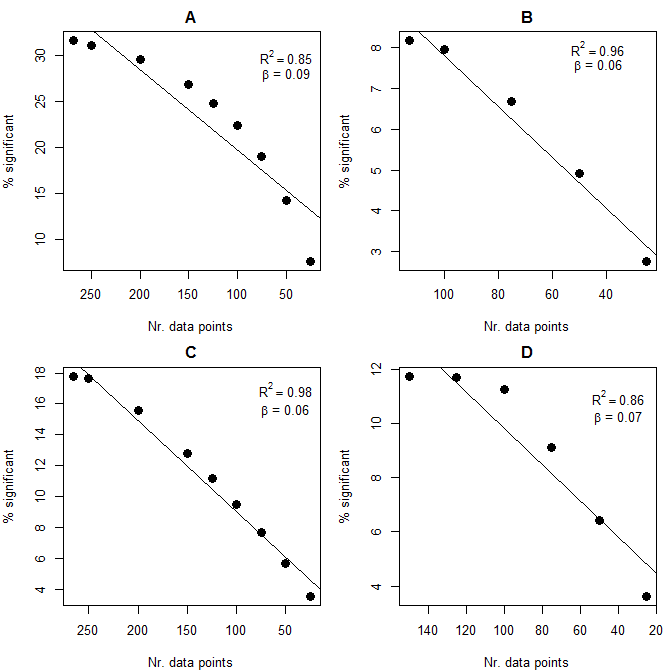


**Figure S10.** Effects of the number of data points used to estimate time series regression models. The y-axes show the proportions of significant interactions relative to the total number of potential interactions (square of interaction matrix dimensions), computed using the full data sets and models using the number of data points specified on the x-axes. The full models (Table 2) have been included in the graphs for interpretability (leftmost points). All the relationships are highly significant (P<0.01 for all regression models). Panels A-D represent results for I1-I4, respectively.


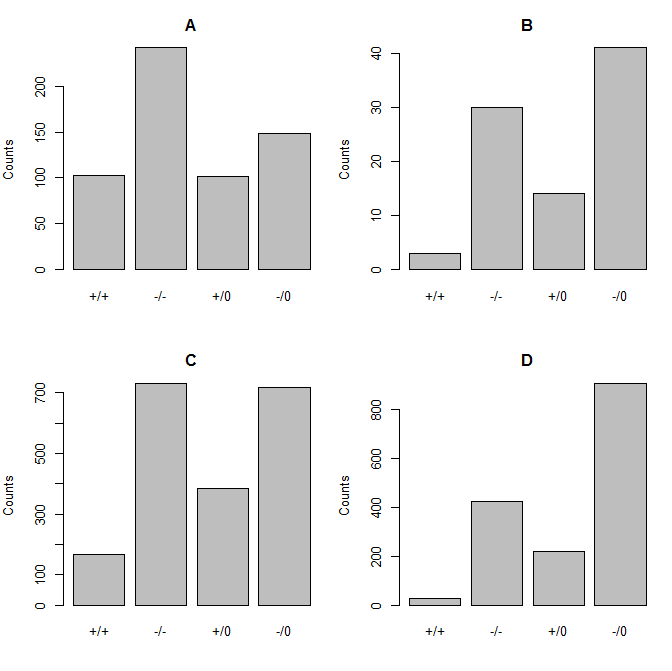


**Figure S11.** Counts of observed cases of cooperation (+/+), competition (-/-), commensalism (+/0) and amensalism (-/0). Panels A-D represent results for I1-I4, respectively.

**
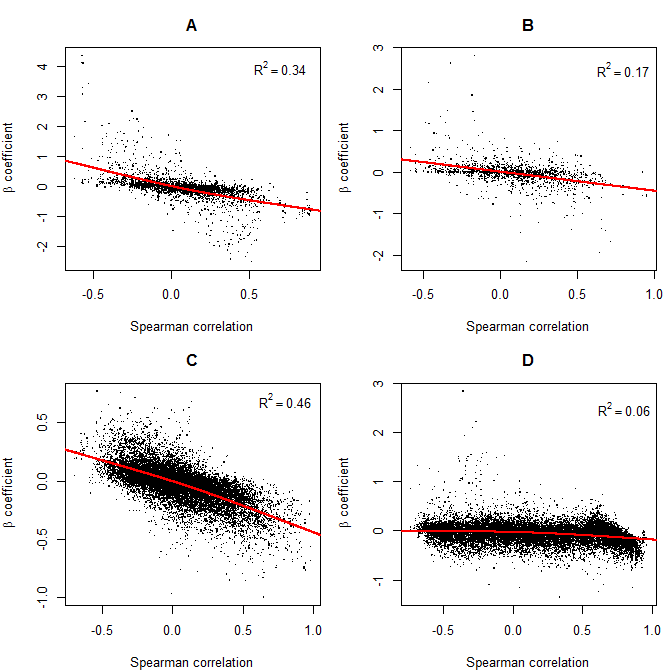
Figure S12.** Relationship between regression coefficients computed from time series models and contemporaneous Spearman correlations representing co-occurrence of observed taxa. Regression lines were computed from generalized additive models using 3 degrees of freedom. All the models except B (I2) showed significant non-linearities. All models were highly significant (P < 0.001). Panels A-D represent results for I1-I4, respectively.


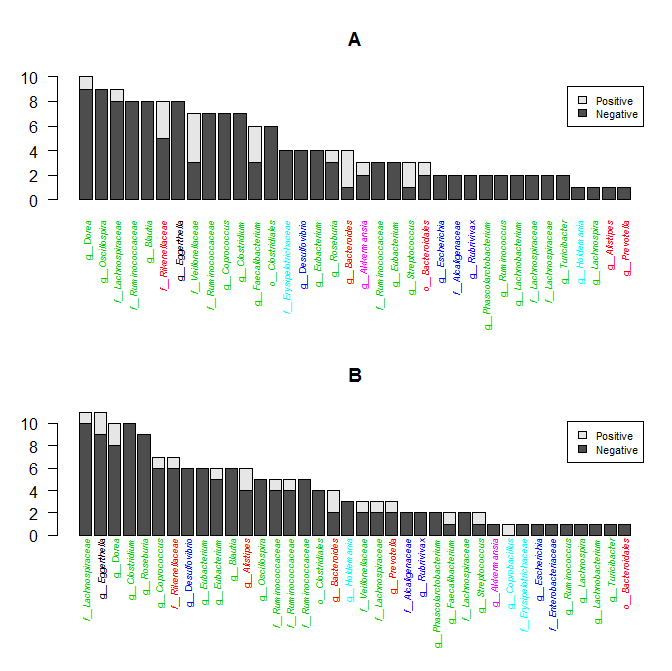
**Figure S13. Genus level degree of connectedness in I2.** The bar charts show the total number of significant negative and positive genus level interactions. Dependent (acted upon) interactions are shown in panel A while independent (acting on) interactions are in panel B. Genus names are coloured according to phylum provenance: Black= *Actinobacteria*, Red= *Bacteroidetes*, Green= *Firmicutes*, Blue=*Proteobacteria*, Light Blue=*Tenericutes*, and Pink=*Verrucomicrobia*.

**
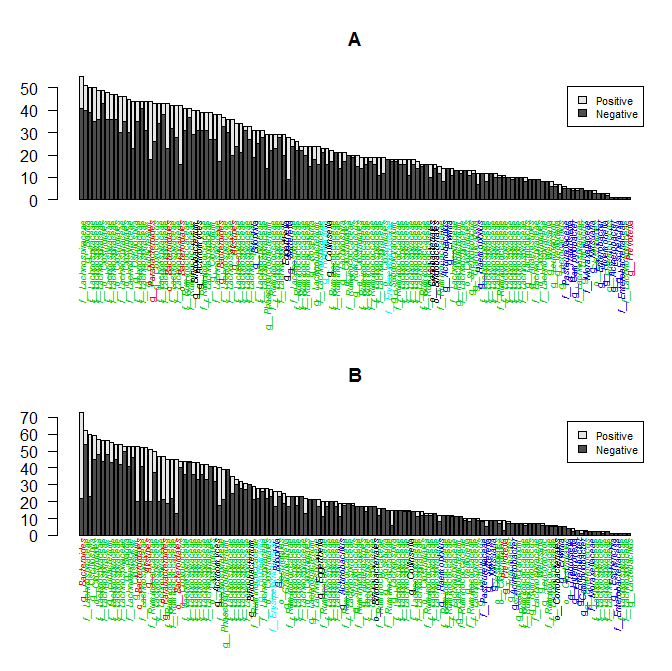
Figure S14. Genus level degree of connectedness in I3.** The bar charts show the total number of significant negative and positive genus level interactions. Dependent (acted upon) interactions are shown in panel A while independent (acting on) interactions are in panel B. Genus names are coloured according to phylum provenance: Black= *Actinobacteria*, Red= *Bacteroidetes*, Green= *Firmicutes*, Blue=*Proteobacteria*, Light Blue=*Tenericutes*, and Pink=*Verrucomicrobia*.

**
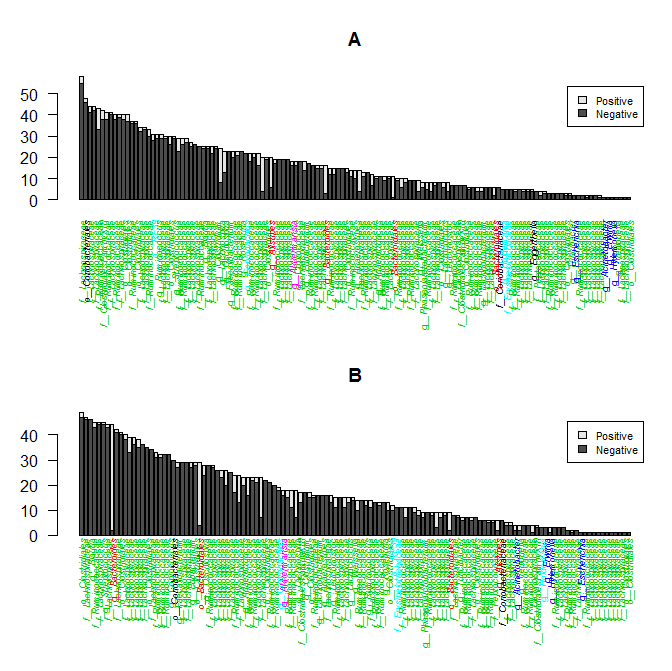
Figure S15. Genus level degree of connectedness in I4.** The bar charts show the total number of significant negative and positive genus level interactions. Dependent (acted upon) interactions are shown in panel A while independent (acting on) interactions are in panel B. Genus names are coloured according to phylum provenance: Black= *Actinobacteria*, Red= *Bacteroidetes*, Green= *Firmicutes*, Blue=*Proteobacteria*, Light Blue=*Tenericutes*, and Pink=*Verrucomicrobia*.

**
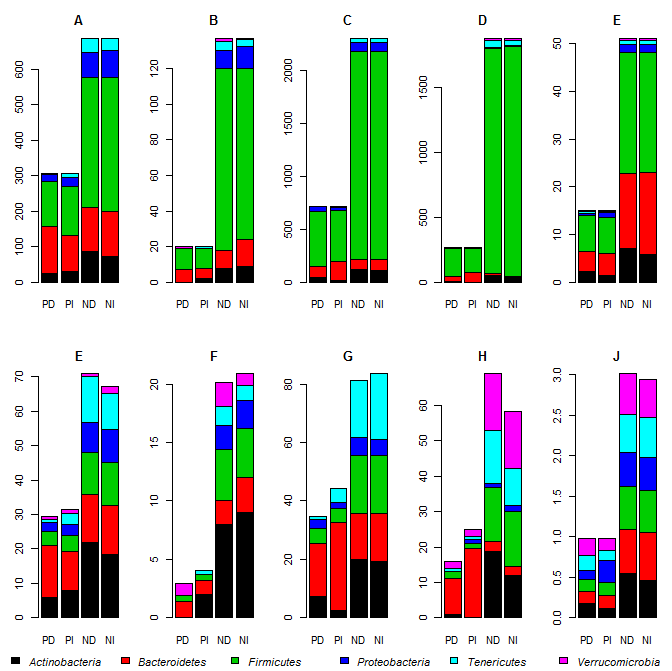
Figure S16. Total and mean connectedness of six main phyla estimated by time series analysis and RE.** Panels A-D show counts of positive and negative interactions significant at the 99% confidence level estimated by time series analysis for I1-4, respectively. Panel E shows summed competition and complementarity indices estimated by RE within the main phyla identified in the time series studies. Panels F-I show the mean connectedness of the phyla, i.e. the counts in A-D divided by the number of observed genera within the respective phyla in each individual. Panel J is the numbers in E divided by the number of observed genera within the respective phyla (see methods).

*
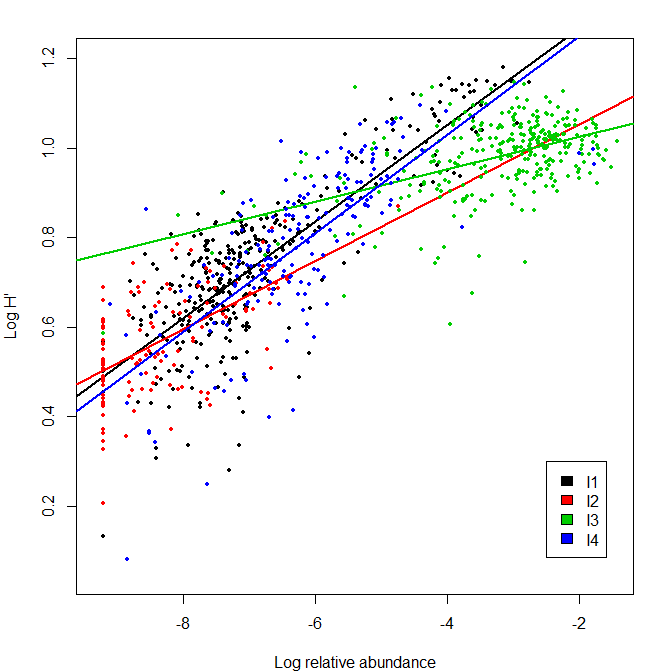
***Figure S17.Within-individual relationship between relative abundance of *Actinobacteria* and total community diversity (Shannon index).** In each individual there was a highly significant positive association (p<<0.001).

**
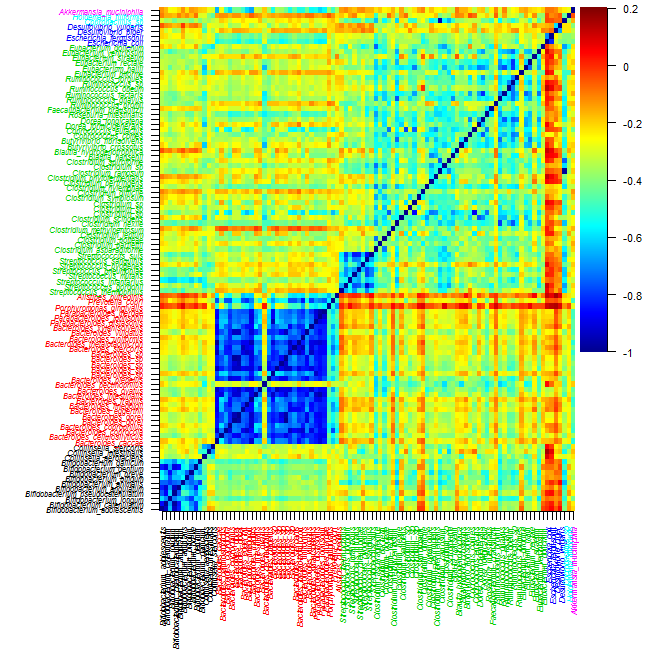
Figure S18. Heat map of net interaction indices estimated by RE.** The plotted values are differences between complementarity and competitive indices. More competitive interactions were observed within than between phyla (p<<0.001). Dependent variables are along the y axis and independent variables along the x axis, i.e. if you follow the column of given genus upward from the x-axis until you reach a coloured cell that cell indicates the effect of the given genus (dependent) on the genus indicated on the y-axis (independent). The colour key on the right-hand side indicates the sign and magnitude of interactions. Species names are coloured according to phylum provenance: Black= *Actinobacteria*, Red= *Bacteroidetes*, Green= *Firmicutes*, Blue=*Proteobacteria*, Light Blue=*Tenericutes*, and Pink=*Verrucomicrobia*.

**
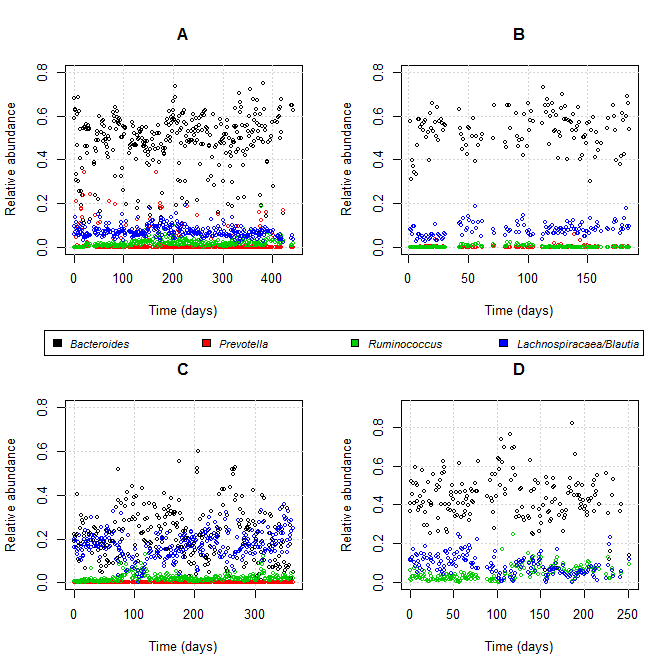
Figure S19. Relative abundances in of genus level OTU’s of *Bacteroides*, *Prevotella*, *Ruminococcus* and family level combinations of *Lachnospiracia* and *Blautia.*** These groups are commonly used for enterotype classification. Panels A-D show data for I1-4, respectively. In I4 *Prevotella* was observed at such low abundances that the group was removed by our filtering criteria.


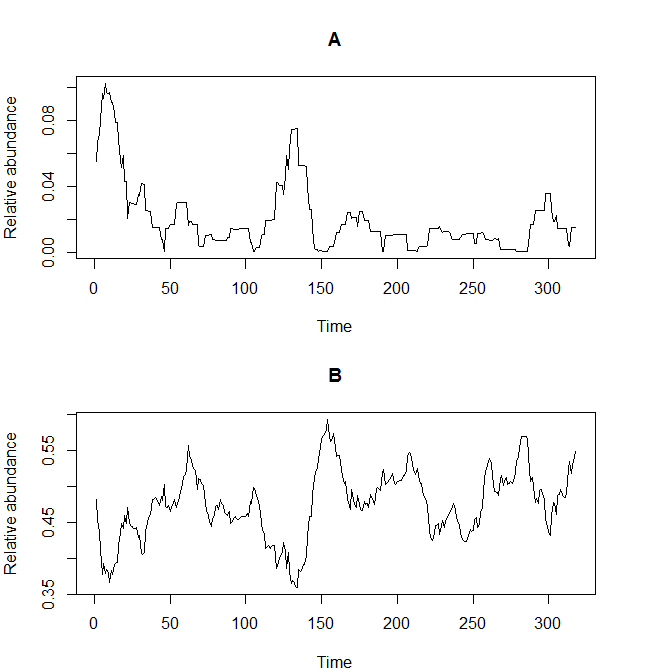


**Figure S20. *Prevotella* (A) and *Bacteroides* (B) relative abundances plotted as smoothed averages along a moving window of 15 data points.** High *Prevotella* abundances are associated with enterotype 2. There was some evidence of temporal clustering of enterotype 2 observations (p=0.015, Wald-Wolfowitz test).
